# *N*-{4-[(3,4-Dimethyl­phen­yl)sulfamo­yl]phen­yl}acetamide

**DOI:** 10.1107/S1600536810029405

**Published:** 2010-07-31

**Authors:** Islam Ullah Khan, Peter John, Saima Khizar, Shahzad Sharif, Edward R. T. Tiekink

**Affiliations:** a Materials Chemistry Laboratory, Department of Chemistry, Government College University, Lahore 54000, Pakistan; b Department of Chemistry, University of Malaya, 50603 Kuala Lumpur, Malaysia

## Abstract

Two independent mol­ecules comprise the asymmetric unit of the title compound, C_16_H_18_N_2_O_3_S. Small but significant twists about the (S)N—C and S—C bonds differentiate the mol­ecules but the most obvious difference is found in the relative orientation of the *meta*-methyl groups, which lie on opposite sides of the mol­ecules. Overall, both mol­ecules adopt a U shape but with significant twisting evident, particularly in the second independent mol­ecule [dihedral angles between benzene rings = 63.90 (13) and 35.78 (11)°]. In the crystal, N—H⋯O hydrogen bonds lead to supra­molecular chains with a tubular topology propagating in [100] and C—H⋯O contacts cross-link the chains.

## Related literature

For background to the anti­microbial activity of sulfonamides, see: Korolkovas, (1988 ▶); Mandell & Sande (1992 ▶). For related structures, see: John *et al.* (2010*a* ▶,*b* ▶).
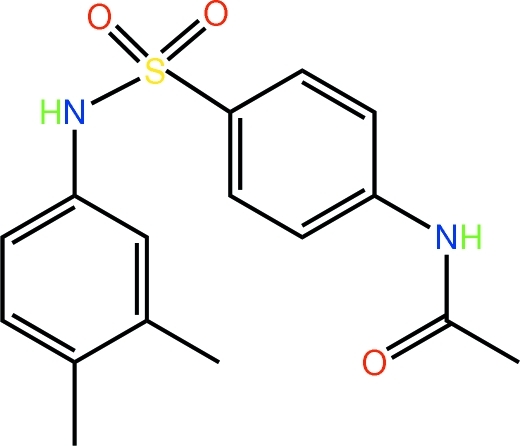


## Experimental

### 

#### Crystal data

C_16_H_18_N_2_O_3_S
*M* *_r_* = 318.40
Triclinic, 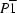

*a* = 8.4317 (3) Å
*b* = 13.6142 (5) Å
*c* = 15.1796 (5) Å
α = 71.340 (1)°
β = 77.136 (1)°
γ = 81.089 (1)°
*V* = 1602.83 (10) Å^3^
*Z* = 4
Mo *K*α radiation
μ = 0.22 mm^−1^
*T* = 293 K
0.27 × 0.11 × 0.08 mm

#### Data collection

Bruker APEXII CCD diffractometer
Absorption correction: multi-scan (*SADABS*; Sheldrick, 1996 ▶) *T*_min_ = 0.817, *T*_max_ = 0.940
27476 measured reflections
7319 independent reflections
5632 reflections with *I* > 2σ(*I*)
*R*_int_ = 0.030

#### Refinement

*R*[*F* ^2^ > 2σ(*F* ^2^)] = 0.046
*wR*(*F* ^2^) = 0.152
*S* = 1.06
7319 reflections
415 parameters
4 restraints
H atoms treated by a mixture of independent and constrained refinement
Δρ_max_ = 0.34 e Å^−3^
Δρ_min_ = −0.39 e Å^−3^

### 

Data collection: *APEX2* (Bruker, 2007 ▶); cell refinement: *SAINT* (Bruker, 2007 ▶); data reduction: *SAINT*; program(s) used to solve structure: *SHELXS97* (Sheldrick, 2008 ▶); program(s) used to refine structure: *SHELXL97* (Sheldrick, 2008 ▶); molecular graphics: *ORTEP-3* (Farrugia, 1997 ▶), *DIAMOND* (Brandenburg, 2006 ▶) and *Qmol* (Gans & Shalloway, 2001 ▶); software used to prepare material for publication: *publCIF* (Westrip, 2010 ▶).

## Supplementary Material

Crystal structure: contains datablocks general, I. DOI: 10.1107/S1600536810029405/hb5572sup1.cif

Structure factors: contains datablocks I. DOI: 10.1107/S1600536810029405/hb5572Isup2.hkl

Additional supplementary materials:  crystallographic information; 3D view; checkCIF report

## Figures and Tables

**Table 1:** Hydrogen-bond geometry (Å, °)

*D*—H⋯*A*	*D*—H	H⋯*A*	*D*⋯*A*	*D*—H⋯*A*
N1—H1n⋯O3^i^	0.86 (2)	2.07 (2)	2.909 (3)	164 (2)
N2—H2n⋯O4^ii^	0.88 (2)	2.08 (2)	2.931 (3)	163 (2)
N3—H3n⋯O1^iii^	0.87 (2)	2.11 (2)	2.960 (3)	166 (2)
N4—H4n⋯O5^iii^	0.87 (2)	2.18 (2)	3.036 (2)	170 (2)
C8—H8b⋯O6^iv^	0.96	2.71	3.640 (3)	163
C6—H6⋯O6^iv^	0.93	2.67	3.541 (3)	156
C16—H16c⋯O6^v^	0.96	2.65	3.477 (3)	145

Symmetry codes: (i) 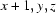; (ii) 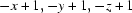; (iii) 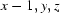; (iv) 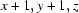; (v) 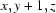.

**Fig. 1.**
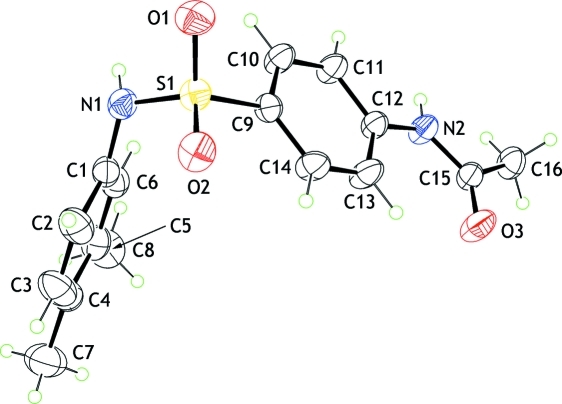
The molecular structure of the first independent molecule in (I) showing displacement ellipsoids at the 35% probability level.

**Fig. 2.**
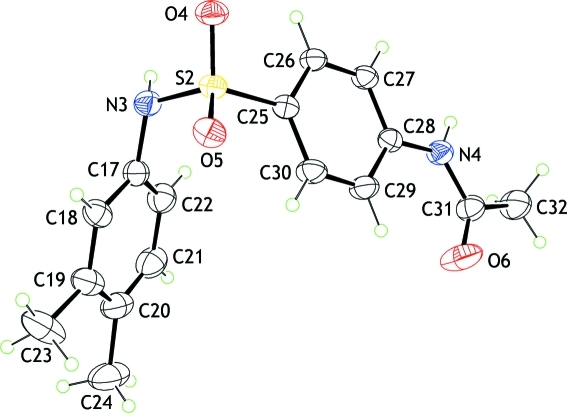
The molecular structure of the second independent molecule in (I) showing displacement ellipsoids at the 35% probability level.

**Fig. 3.**
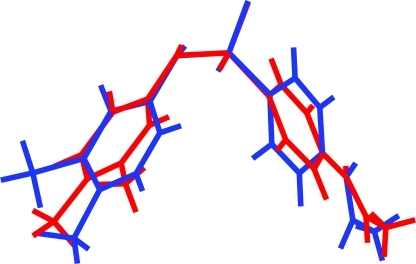
Overlay diagram of the first independent molecule (shown in red) and the second independent molecule (shown in blue).

**Fig. 4.**
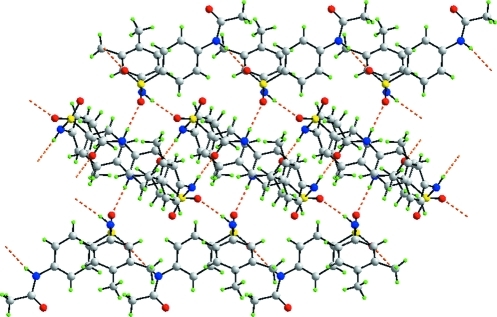
Linear supramolecular chain along [100] in (I) mediated by N–H···O hydrogen bonding, shown as orange dashed lines.

## supplementary crystallographic information

### Comment

Sulfonamides related to the title compound exhibit anti-microbial activity (Korolkovas, 1988; Mandell & Sande, 1992). In connection with on-going structural studies of sulfonamides containing acetamide residues (John *et al.*, 2010*a*; John *et al.*, 2010*b*), the crystal and molecular structure of the title compound, (I), was investigated.

Two independent molecules comprise the crystallographic asymmetric unit of (I). There are non-chemically significant differences between the two molecules with the first, Fig. 1, being almost super-imposable upon the second, Fig. 2, but with twists evident about the (S)N–C and S–C bonds, Fig. 3. About the former, the differences are quantified in the S1–N1–C1–C2 and S2–N3–C17–C22 torsion angles of -107.5 (2) and -93.8 (2) °, respectively. About the S–C bond, the twists are evident in the O1–S1–C9–C10 and O4–S2–C25–C26 torsion angles of -44.3 (2) and -25.36 (19) °, respectively. In each case, the acetamide group is co-planar with the benzene ring to which it is bonded [C12–N2–C15–O3 = 0.5 (4) ° and C28–N4–C31–O6 = -3.2 (4) °]. The major difference relates to the relative disposition of the *meta*-methyl group which effectively resides on opposite sides in the two molecules, Fig. 3. Within each molecule, the benzene molecules are orientated in the same direction and form dihedral angles of 63.90 (13) ° (first molecule) and 35.78 (11) ° so that overall the molecules have a U-shaped but with significant twisting, in particular for the second independent molecule.

The crystal packing is dominated by N–H···O hydrogen bonds whereby each of the amide-N atoms forms an interaction with a sulfamoyl-O atom, Table 1. The sulfamoyl-N–H forms an interaction with an amide-O in the case of N1 but with a sulfamoyl-O atom in the case of N4, Table 1. The result of the hydrogen bonding is the formation of a supramolecular chain along the *a* axis with a tubular topology, Fig. 4. Perhaps surprisingly, the hydrogen bonding scheme does not involve the amide-O6 atom, which lies to the periphery of the chain, Fig. 4. However, the O6 atom forms a very short intramolecular C–H···O contact [H···O = 2.22 Å] and forms three further C–H···O contacts less than 2.72 Å [shortest = 2.65 Å with H16*c*^i^ where *i*: *x*, -1 + *y*, *z*], thereby providing links between the supramolecular chains.

### Experimental

To 3,4-dimethyl aniline (242 mg, 2 mmol) in distilled water (10 ml) was added 4-acetamido benzene sulfonyl chloride (467 mg, 2 mmol) with stirring at room temperature while maintaining the pH of the reaction mixture at pH 8 using 3% sodium carbonate. The progress of the reaction was monitored by TLC. The precipitate formed was washed with water, dried and crystallized from a methanol/ethyl acetate mixture (50:50 V/V) to yield light-orange blocks of (I).

### Refinement

The C-bound H atoms were geometrically placed (C–H = 0.93–0.97 Å) and refined as riding with *U_iso_*(H) = 1.2–1.5*U_eq_*(C). The N-bound H atoms were refined with the distance restraint N–H = 0.88±0.01 Å, and with *U_iso_*(H) = 1.2*U_eq_*(N). In the final refinement, six low angle reflections evidently affected by the beam stop were omitted, *i.e.* 0 1 1; 1 1 1; 0 0 1; 0 1 0; 0 1 1; and 1 1 0.

### Crystal data

**Table:**

C_16_H_18_N_2_O_3_S	*Z* = 4
*M**_r_* = 318.40	*F*(000) = 672
Triclinic, *P*1	*D*_x_ = 1.319 Mg m^−^^3^
Hall symbol: -P 1	Mo *K*α radiation, λ = 0.71073 Å
*a* = 8.4317 (3) Å	Cell parameters from 9916 reflections
*b* = 13.6142 (5) Å	θ = 2.5–28.2°
*c* = 15.1796 (5) Å	µ = 0.22 mm^−^^1^
α = 71.340 (1)°	*T* = 293 K
β = 77.136 (1)°	Prism, light-orange
γ = 81.089 (1)°	0.27 × 0.11 × 0.08 mm
*V* = 1602.83 (10) Å^3^	

### Data collection

**Table:**

Bruker APEXII CCD diffractometer	7319 independent reflections
Radiation source: fine-focus sealed tube	5632 reflections with *I* > 2σ(*I*)
graphite	*R*_int_ = 0.030
φ and ω scans	θ_max_ = 27.5°, θ_min_ = 2.5°
Absorption correction: multi-scan (*SADABS*; Sheldrick, 1996)	*h* = −10→10
*T*_min_ = 0.817, *T*_max_ = 0.940	*k* = −17→17
27476 measured reflections	*l* = −19→19

### Refinement

**Table:**

Refinement on *F*^2^	Primary atom site location: structure-invariant direct methods
Least-squares matrix: full	Secondary atom site location: difference Fourier map
*R*[*F*^2^ > 2σ(*F*^2^)] = 0.046	Hydrogen site location: inferred from neighbouring sites
*wR*(*F*^2^) = 0.152	H atoms treated by a mixture of independent and constrained refinement
*S* = 1.06	*w* = 1/[σ^2^(*F*_o_^2^) + (0.079*P*)^2^ + 0.545*P*] where *P* = (*F*_o_^2^ + 2*F*_c_^2^)/3
7319 reflections	(Δ/σ)_max_ = 0.001
415 parameters	Δρ_max_ = 0.34 e Å^−^^3^
4 restraints	Δρ_min_ = −0.39 e Å^−^^3^

### Special details

**Table:**

Geometry. All s.u.'s (except the s.u. in the dihedral angle between two l.s. planes) are estimated using the full covariance matrix. The cell s.u.'s are taken into account individually in the estimation of s.u.'s in distances, angles and torsion angles; correlations between s.u.'s in cell parameters are only used when they are defined by crystal symmetry. An approximate (isotropic) treatment of cell s.u.'s is used for estimating s.u.'s involving l.s. planes.
Refinement. Refinement of *F*^2^ against ALL reflections. The weighted *R*-factor *wR* and goodness of fit *S* are based on *F*^2^, conventional *R*-factors *R* are based on *F*, with *F* set to zero for negative *F*^2^. The threshold expression of *F*^2^ > 2σ(*F*^2^) is used only for calculating *R*-factors(gt) *etc*. and is not relevant to the choice of reflections for refinement. *R*-factors based on *F*^2^ are statistically about twice as large as those based on *F*, and *R*- factors based on ALL data will be even larger.

### Fractional atomic coordinates and isotropic or equivalent isotropic displacement parameters (Å^2^)

**Table:**

	*x*	*y*	*z*	*U*_iso_*/*U*_eq_	
S1	0.98322 (6)	0.29377 (4)	0.73537 (4)	0.04718 (16)	
O1	1.1132 (2)	0.28964 (14)	0.65701 (12)	0.0646 (5)	
O2	0.9185 (2)	0.20011 (12)	0.79683 (13)	0.0621 (4)	
O3	0.2213 (2)	0.51502 (15)	0.64850 (14)	0.0716 (5)	
N1	1.0577 (2)	0.34788 (15)	0.79690 (13)	0.0490 (4)	
H1N	1.105 (3)	0.4029 (13)	0.7617 (15)	0.059*	
N2	0.4537 (2)	0.59093 (14)	0.57736 (14)	0.0516 (5)	
H2N	0.488 (3)	0.6506 (12)	0.5403 (15)	0.062*	
C1	0.9606 (3)	0.36318 (18)	0.88249 (15)	0.0479 (5)	
C2	0.9398 (4)	0.2785 (2)	0.96293 (18)	0.0641 (6)	
H2	0.9835	0.2122	0.9603	0.077*	
C3	0.8541 (4)	0.2932 (2)	1.04648 (19)	0.0732 (8)	
H3	0.8399	0.2361	1.1003	0.088*	
C4	0.7889 (3)	0.3900 (2)	1.05273 (18)	0.0638 (6)	
C5	0.8081 (3)	0.4762 (2)	0.97240 (19)	0.0595 (6)	
C6	0.8955 (3)	0.46140 (19)	0.88680 (17)	0.0540 (5)	
H6	0.9097	0.5181	0.8326	0.065*	
C7	0.6971 (5)	0.4053 (3)	1.1468 (2)	0.0939 (10)	
H7A	0.6751	0.3387	1.1917	0.141*	
H7B	0.5959	0.4470	1.1376	0.141*	
H7C	0.7625	0.4397	1.1700	0.141*	
C8	0.7413 (4)	0.5840 (2)	0.9746 (2)	0.0844 (9)	
H8A	0.6241	0.5881	0.9881	0.127*	
H8B	0.7773	0.6328	0.9143	0.127*	
H8C	0.7796	0.6003	1.0228	0.127*	
C9	0.8222 (2)	0.37923 (16)	0.69024 (14)	0.0417 (4)	
C10	0.8574 (3)	0.47298 (17)	0.62245 (17)	0.0524 (5)	
H10	0.9649	0.4899	0.6015	0.063*	
C11	0.7335 (3)	0.54057 (17)	0.58647 (17)	0.0522 (5)	
H11	0.7574	0.6033	0.5407	0.063*	
C12	0.5724 (2)	0.51646 (16)	0.61760 (15)	0.0433 (4)	
C13	0.5385 (3)	0.42172 (19)	0.68438 (18)	0.0591 (6)	
H13	0.4314	0.4040	0.7048	0.071*	
C14	0.6637 (3)	0.35388 (18)	0.72046 (17)	0.0546 (6)	
H14	0.6407	0.2905	0.7656	0.065*	
C15	0.2910 (2)	0.58790 (17)	0.59298 (15)	0.0462 (5)	
C16	0.2010 (3)	0.6818 (2)	0.53545 (18)	0.0591 (6)	
H16A	0.1901	0.6702	0.4780	0.089*	
H16B	0.2607	0.7415	0.5204	0.089*	
H16C	0.0945	0.6939	0.5712	0.089*	
S2	0.43431 (5)	0.11151 (4)	0.58705 (3)	0.03881 (14)	
O4	0.42248 (18)	0.20348 (12)	0.50888 (10)	0.0522 (4)	
O5	0.57822 (16)	0.04038 (12)	0.58419 (10)	0.0478 (3)	
O6	−0.0669 (2)	−0.28130 (14)	0.73484 (17)	0.0821 (6)	
N3	0.4199 (2)	0.15276 (13)	0.67873 (12)	0.0430 (4)	
H3N	0.3329 (19)	0.1964 (15)	0.6800 (16)	0.052*	
N4	−0.1488 (2)	−0.11535 (14)	0.65969 (13)	0.0449 (4)	
H4N	−0.2356 (19)	−0.0768 (16)	0.6428 (16)	0.054*	
C17	0.4346 (2)	0.07454 (16)	0.76766 (14)	0.0419 (4)	
C18	0.5893 (3)	0.03908 (17)	0.78749 (14)	0.0467 (5)	
H18	0.6796	0.0685	0.7452	0.056*	
C19	0.6119 (3)	−0.03979 (19)	0.86957 (16)	0.0563 (6)	
C20	0.4745 (4)	−0.08221 (18)	0.93320 (16)	0.0610 (6)	
C21	0.3204 (4)	−0.0419 (2)	0.91310 (17)	0.0626 (6)	
H21	0.2288	−0.0678	0.9566	0.075*	
C22	0.2991 (3)	0.03525 (19)	0.83092 (16)	0.0540 (5)	
H22	0.1948	0.0603	0.8185	0.065*	
C23	0.7826 (4)	−0.0759 (3)	0.8881 (2)	0.0831 (10)	
H23A	0.7900	−0.0700	0.9484	0.125*	
H23B	0.8586	−0.0334	0.8393	0.125*	
H23C	0.8081	−0.1472	0.8883	0.125*	
C24	0.4914 (5)	−0.1688 (2)	1.02311 (19)	0.0908 (11)	
H24A	0.3848	−0.1860	1.0587	0.136*	
H24B	0.5491	−0.1466	1.0602	0.136*	
H24C	0.5509	−0.2290	1.0076	0.136*	
C25	0.2646 (2)	0.04302 (15)	0.60532 (13)	0.0374 (4)	
C26	0.1210 (2)	0.09493 (15)	0.57695 (14)	0.0401 (4)	
H26	0.1155	0.1660	0.5456	0.048*	
C27	−0.0130 (2)	0.04060 (15)	0.59549 (14)	0.0414 (4)	
H27	−0.1093	0.0752	0.5763	0.050*	
C28	−0.0064 (2)	−0.06551 (15)	0.64266 (13)	0.0375 (4)	
C29	0.1378 (3)	−0.11701 (17)	0.67113 (16)	0.0481 (5)	
H29	0.1436	−0.1880	0.7027	0.058*	
C30	0.2719 (2)	−0.06227 (16)	0.65223 (16)	0.0479 (5)	
H30	0.3684	−0.0966	0.6713	0.058*	
C31	−0.1732 (3)	−0.21692 (17)	0.70512 (16)	0.0500 (5)	
C32	−0.3442 (3)	−0.2432 (2)	0.7171 (2)	0.0676 (7)	
H32A	−0.3471	−0.3174	0.7390	0.101*	
H32B	−0.3802	−0.2160	0.6575	0.101*	
H32C	−0.4150	−0.2130	0.7624	0.101*	

### Atomic displacement parameters (Å^2^)

**Table:**

	*U*^11^	*U*^22^	*U*^33^	*U*^12^	*U*^13^	*U*^23^
S1	0.0455 (3)	0.0374 (3)	0.0539 (3)	0.0065 (2)	−0.0116 (2)	−0.0101 (2)
O1	0.0577 (9)	0.0618 (11)	0.0668 (11)	0.0172 (8)	−0.0044 (8)	−0.0239 (9)
O2	0.0679 (10)	0.0357 (8)	0.0770 (11)	−0.0009 (7)	−0.0216 (9)	−0.0051 (8)
O3	0.0444 (8)	0.0658 (11)	0.0858 (13)	−0.0150 (8)	−0.0131 (8)	0.0089 (10)
N1	0.0439 (9)	0.0468 (10)	0.0514 (10)	−0.0044 (7)	−0.0128 (8)	−0.0049 (8)
N2	0.0404 (9)	0.0415 (10)	0.0610 (11)	−0.0075 (7)	−0.0141 (8)	0.0059 (8)
C1	0.0462 (11)	0.0495 (12)	0.0479 (11)	−0.0076 (9)	−0.0156 (9)	−0.0079 (9)
C2	0.0833 (18)	0.0505 (14)	0.0547 (14)	−0.0087 (12)	−0.0202 (12)	−0.0041 (11)
C3	0.102 (2)	0.0596 (17)	0.0513 (14)	−0.0204 (15)	−0.0161 (14)	0.0007 (12)
C4	0.0640 (15)	0.0708 (17)	0.0557 (14)	−0.0198 (12)	−0.0127 (11)	−0.0101 (12)
C5	0.0582 (13)	0.0540 (14)	0.0721 (16)	−0.0057 (11)	−0.0218 (12)	−0.0196 (12)
C6	0.0547 (12)	0.0493 (13)	0.0562 (13)	−0.0065 (10)	−0.0187 (10)	−0.0065 (10)
C7	0.104 (3)	0.111 (3)	0.0642 (18)	−0.030 (2)	0.0025 (17)	−0.0253 (18)
C8	0.101 (2)	0.0665 (19)	0.088 (2)	0.0055 (16)	−0.0203 (18)	−0.0299 (16)
C9	0.0418 (10)	0.0380 (10)	0.0428 (10)	0.0009 (8)	−0.0108 (8)	−0.0085 (8)
C10	0.0392 (10)	0.0464 (12)	0.0626 (14)	−0.0084 (9)	−0.0101 (9)	−0.0015 (10)
C11	0.0431 (11)	0.0416 (12)	0.0596 (13)	−0.0102 (9)	−0.0114 (9)	0.0061 (10)
C12	0.0384 (9)	0.0402 (11)	0.0469 (11)	−0.0039 (8)	−0.0107 (8)	−0.0047 (9)
C13	0.0392 (10)	0.0521 (13)	0.0684 (15)	−0.0094 (9)	−0.0076 (10)	0.0073 (11)
C14	0.0486 (11)	0.0432 (12)	0.0557 (13)	−0.0061 (9)	−0.0064 (10)	0.0063 (10)
C15	0.0397 (10)	0.0475 (12)	0.0509 (12)	−0.0060 (8)	−0.0108 (8)	−0.0111 (10)
C16	0.0473 (12)	0.0582 (14)	0.0693 (15)	0.0021 (10)	−0.0215 (11)	−0.0114 (12)
S2	0.0337 (2)	0.0399 (3)	0.0370 (3)	−0.00307 (18)	−0.00693 (17)	−0.0032 (2)
O4	0.0509 (8)	0.0477 (9)	0.0472 (8)	−0.0108 (6)	−0.0123 (6)	0.0059 (7)
O5	0.0347 (7)	0.0563 (9)	0.0480 (8)	0.0032 (6)	−0.0056 (6)	−0.0141 (7)
O6	0.0568 (10)	0.0438 (10)	0.1222 (17)	−0.0065 (8)	−0.0184 (10)	0.0100 (10)
N3	0.0425 (9)	0.0375 (9)	0.0483 (9)	0.0003 (7)	−0.0121 (7)	−0.0109 (7)
N4	0.0395 (8)	0.0405 (10)	0.0530 (10)	−0.0030 (7)	−0.0140 (7)	−0.0083 (8)
C17	0.0471 (10)	0.0400 (11)	0.0396 (10)	−0.0025 (8)	−0.0088 (8)	−0.0134 (8)
C18	0.0494 (11)	0.0511 (12)	0.0402 (10)	0.0029 (9)	−0.0105 (8)	−0.0165 (9)
C19	0.0755 (15)	0.0512 (13)	0.0453 (12)	0.0171 (11)	−0.0212 (11)	−0.0224 (10)
C20	0.102 (2)	0.0404 (12)	0.0401 (11)	−0.0001 (12)	−0.0145 (12)	−0.0133 (10)
C21	0.0804 (17)	0.0556 (14)	0.0489 (13)	−0.0188 (13)	−0.0002 (12)	−0.0134 (11)
C22	0.0533 (12)	0.0550 (13)	0.0534 (13)	−0.0089 (10)	−0.0069 (10)	−0.0157 (11)
C23	0.089 (2)	0.100 (2)	0.0619 (16)	0.0414 (18)	−0.0383 (15)	−0.0322 (16)
C24	0.160 (3)	0.0508 (16)	0.0530 (15)	0.0002 (18)	−0.0255 (18)	−0.0038 (13)
C25	0.0364 (9)	0.0375 (10)	0.0358 (9)	−0.0003 (7)	−0.0069 (7)	−0.0085 (8)
C26	0.0414 (9)	0.0333 (10)	0.0414 (10)	0.0004 (7)	−0.0111 (8)	−0.0048 (8)
C27	0.0369 (9)	0.0387 (10)	0.0462 (11)	0.0024 (7)	−0.0134 (8)	−0.0081 (8)
C28	0.0389 (9)	0.0382 (10)	0.0348 (9)	−0.0016 (7)	−0.0074 (7)	−0.0103 (8)
C29	0.0466 (11)	0.0351 (10)	0.0569 (12)	−0.0011 (8)	−0.0154 (9)	−0.0032 (9)
C30	0.0388 (10)	0.0400 (11)	0.0587 (13)	0.0042 (8)	−0.0162 (9)	−0.0048 (9)
C31	0.0481 (11)	0.0446 (12)	0.0527 (12)	−0.0085 (9)	−0.0062 (9)	−0.0081 (10)
C32	0.0576 (14)	0.0593 (15)	0.0813 (18)	−0.0187 (12)	−0.0171 (12)	−0.0057 (13)

### Geometric parameters (Å, °)

**Table:**

S1—O2	1.4219 (17)	S2—O4	1.4315 (14)
S1—O1	1.4363 (17)	S2—O5	1.4322 (14)
S1—N1	1.629 (2)	S2—N3	1.6342 (18)
S1—C9	1.755 (2)	S2—C25	1.7510 (19)
O3—C15	1.210 (3)	O6—C31	1.208 (3)
N1—C1	1.431 (3)	N3—C17	1.444 (3)
N1—H1N	0.866 (10)	N3—H3N	0.870 (10)
N2—C15	1.344 (3)	N4—C31	1.358 (3)
N2—C12	1.402 (3)	N4—C28	1.404 (2)
N2—H2N	0.876 (10)	N4—H4N	0.870 (10)
C1—C6	1.381 (3)	C17—C22	1.374 (3)
C1—C2	1.385 (3)	C17—C18	1.382 (3)
C2—C3	1.371 (4)	C18—C19	1.388 (3)
C2—H2	0.9300	C18—H18	0.9300
C3—C4	1.371 (4)	C19—C20	1.400 (4)
C3—H3	0.9300	C19—C23	1.505 (4)
C4—C5	1.394 (4)	C20—C21	1.390 (4)
C4—C7	1.525 (4)	C20—C24	1.509 (3)
C5—C6	1.402 (4)	C21—C22	1.376 (3)
C5—C8	1.497 (4)	C21—H21	0.9300
C6—H6	0.9300	C22—H22	0.9300
C7—H7A	0.9600	C23—H23A	0.9600
C7—H7B	0.9600	C23—H23B	0.9600
C7—H7C	0.9600	C23—H23C	0.9600
C8—H8A	0.9600	C24—H24A	0.9600
C8—H8B	0.9600	C24—H24B	0.9600
C8—H8C	0.9600	C24—H24C	0.9600
C9—C14	1.372 (3)	C25—C30	1.380 (3)
C9—C10	1.385 (3)	C25—C26	1.386 (3)
C10—C11	1.368 (3)	C26—C27	1.374 (3)
C10—H10	0.9300	C26—H26	0.9300
C11—C12	1.389 (3)	C27—C28	1.391 (3)
C11—H11	0.9300	C27—H27	0.9300
C12—C13	1.386 (3)	C28—C29	1.390 (3)
C13—C14	1.378 (3)	C29—C30	1.377 (3)
C13—H13	0.9300	C29—H29	0.9300
C14—H14	0.9300	C30—H30	0.9300
C15—C16	1.501 (3)	C31—C32	1.497 (3)
C16—H16A	0.9600	C32—H32A	0.9600
C16—H16B	0.9600	C32—H32B	0.9600
C16—H16C	0.9600	C32—H32C	0.9600
			
O2—S1—O1	119.55 (11)	O4—S2—O5	119.22 (9)
O2—S1—N1	108.66 (10)	O4—S2—N3	105.44 (10)
O1—S1—N1	104.34 (11)	O5—S2—N3	107.14 (9)
O2—S1—C9	108.30 (10)	O4—S2—C25	108.13 (9)
O1—S1—C9	107.73 (10)	O5—S2—C25	108.38 (9)
N1—S1—C9	107.70 (10)	N3—S2—C25	108.06 (9)
C1—N1—S1	119.80 (14)	C17—N3—S2	116.87 (13)
C1—N1—H1N	111.8 (17)	C17—N3—H3N	115.2 (15)
S1—N1—H1N	112.1 (17)	S2—N3—H3N	107.0 (16)
C15—N2—C12	129.42 (18)	C31—N4—C28	128.34 (17)
C15—N2—H2N	114.2 (18)	C31—N4—H4N	114.1 (16)
C12—N2—H2N	116.2 (18)	C28—N4—H4N	117.4 (16)
C6—C1—C2	120.1 (2)	C22—C17—C18	120.5 (2)
C6—C1—N1	120.9 (2)	C22—C17—N3	121.19 (19)
C2—C1—N1	118.9 (2)	C18—C17—N3	118.32 (18)
C3—C2—C1	119.4 (3)	C17—C18—C19	121.0 (2)
C3—C2—H2	120.3	C17—C18—H18	119.5
C1—C2—H2	120.3	C19—C18—H18	119.5
C4—C3—C2	121.7 (2)	C18—C19—C20	118.8 (2)
C4—C3—H3	119.2	C18—C19—C23	119.1 (2)
C2—C3—H3	119.2	C20—C19—C23	122.1 (2)
C3—C4—C5	119.7 (2)	C21—C20—C19	118.8 (2)
C3—C4—C7	120.9 (3)	C21—C20—C24	120.0 (3)
C5—C4—C7	119.3 (3)	C19—C20—C24	121.2 (3)
C4—C5—C6	118.7 (2)	C22—C21—C20	122.1 (2)
C4—C5—C8	122.4 (3)	C22—C21—H21	119.0
C6—C5—C8	118.8 (2)	C20—C21—H21	119.0
C1—C6—C5	120.4 (2)	C17—C22—C21	118.8 (2)
C1—C6—H6	119.8	C17—C22—H22	120.6
C5—C6—H6	119.8	C21—C22—H22	120.6
C4—C7—H7A	109.5	C19—C23—H23A	109.5
C4—C7—H7B	109.5	C19—C23—H23B	109.5
H7A—C7—H7B	109.5	H23A—C23—H23B	109.5
C4—C7—H7C	109.5	C19—C23—H23C	109.5
H7A—C7—H7C	109.5	H23A—C23—H23C	109.5
H7B—C7—H7C	109.5	H23B—C23—H23C	109.5
C5—C8—H8A	109.5	C20—C24—H24A	109.5
C5—C8—H8B	109.5	C20—C24—H24B	109.5
H8A—C8—H8B	109.5	H24A—C24—H24B	109.5
C5—C8—H8C	109.5	C20—C24—H24C	109.5
H8A—C8—H8C	109.5	H24A—C24—H24C	109.5
H8B—C8—H8C	109.5	H24B—C24—H24C	109.5
C14—C9—C10	120.07 (18)	C30—C25—C26	120.04 (18)
C14—C9—S1	121.03 (16)	C30—C25—S2	119.56 (14)
C10—C9—S1	118.90 (15)	C26—C25—S2	120.33 (15)
C11—C10—C9	119.73 (19)	C27—C26—C25	119.47 (18)
C11—C10—H10	120.1	C27—C26—H26	120.3
C9—C10—H10	120.1	C25—C26—H26	120.3
C10—C11—C12	120.71 (19)	C26—C27—C28	120.75 (17)
C10—C11—H11	119.6	C26—C27—H27	119.6
C12—C11—H11	119.6	C28—C27—H27	119.6
C13—C12—C11	119.18 (19)	C29—C28—C27	119.49 (18)
C13—C12—N2	124.18 (18)	C29—C28—N4	123.14 (18)
C11—C12—N2	116.63 (18)	C27—C28—N4	117.37 (16)
C14—C13—C12	119.9 (2)	C30—C29—C28	119.55 (19)
C14—C13—H13	120.1	C30—C29—H29	120.2
C12—C13—H13	120.1	C28—C29—H29	120.2
C9—C14—C13	120.4 (2)	C29—C30—C25	120.70 (18)
C9—C14—H14	119.8	C29—C30—H30	119.6
C13—C14—H14	119.8	C25—C30—H30	119.6
O3—C15—N2	123.0 (2)	O6—C31—N4	123.2 (2)
O3—C15—C16	122.0 (2)	O6—C31—C32	122.2 (2)
N2—C15—C16	114.91 (19)	N4—C31—C32	114.7 (2)
C15—C16—H16A	109.5	C31—C32—H32A	109.5
C15—C16—H16B	109.5	C31—C32—H32B	109.5
H16A—C16—H16B	109.5	H32A—C32—H32B	109.5
C15—C16—H16C	109.5	C31—C32—H32C	109.5
H16A—C16—H16C	109.5	H32A—C32—H32C	109.5
H16B—C16—H16C	109.5	H32B—C32—H32C	109.5
			
O2—S1—N1—C1	−50.44 (18)	O4—S2—N3—C17	−177.69 (14)
O1—S1—N1—C1	−179.04 (16)	O5—S2—N3—C17	−49.72 (16)
C9—S1—N1—C1	66.66 (18)	C25—S2—N3—C17	66.87 (16)
S1—N1—C1—C6	−107.5 (2)	S2—N3—C17—C22	−93.8 (2)
S1—N1—C1—C2	75.9 (2)	S2—N3—C17—C18	85.6 (2)
C6—C1—C2—C3	0.1 (4)	C22—C17—C18—C19	2.7 (3)
N1—C1—C2—C3	176.6 (2)	N3—C17—C18—C19	−176.69 (19)
C1—C2—C3—C4	−0.4 (4)	C17—C18—C19—C20	−0.9 (3)
C2—C3—C4—C5	0.6 (4)	C17—C18—C19—C23	180.0 (2)
C2—C3—C4—C7	−178.7 (3)	C18—C19—C20—C21	−1.7 (3)
C3—C4—C5—C6	−0.5 (4)	C23—C19—C20—C21	177.3 (2)
C7—C4—C5—C6	178.8 (3)	C18—C19—C20—C24	179.4 (2)
C3—C4—C5—C8	−179.4 (3)	C23—C19—C20—C24	−1.6 (4)
C7—C4—C5—C8	−0.2 (4)	C19—C20—C21—C22	2.8 (4)
C2—C1—C6—C5	0.0 (3)	C24—C20—C21—C22	−178.3 (2)
N1—C1—C6—C5	−176.5 (2)	C18—C17—C22—C21	−1.7 (3)
C4—C5—C6—C1	0.2 (3)	N3—C17—C22—C21	177.7 (2)
C8—C5—C6—C1	179.2 (2)	C20—C21—C22—C17	−1.1 (4)
O2—S1—C9—C14	4.5 (2)	O4—S2—C25—C30	157.72 (17)
O1—S1—C9—C14	135.1 (2)	O5—S2—C25—C30	27.17 (19)
N1—S1—C9—C14	−112.9 (2)	N3—S2—C25—C30	−88.61 (18)
O2—S1—C9—C10	−174.89 (18)	O4—S2—C25—C26	−25.36 (19)
O1—S1—C9—C10	−44.3 (2)	O5—S2—C25—C26	−155.90 (16)
N1—S1—C9—C10	67.8 (2)	N3—S2—C25—C26	88.32 (17)
C14—C9—C10—C11	0.6 (4)	C30—C25—C26—C27	−0.3 (3)
S1—C9—C10—C11	−179.98 (19)	S2—C25—C26—C27	−177.26 (15)
C9—C10—C11—C12	0.4 (4)	C25—C26—C27—C28	0.3 (3)
C10—C11—C12—C13	−1.4 (4)	C26—C27—C28—C29	−0.1 (3)
C10—C11—C12—N2	179.4 (2)	C26—C27—C28—N4	179.47 (18)
C15—N2—C12—C13	−0.1 (4)	C31—N4—C28—C29	0.6 (3)
C15—N2—C12—C11	179.1 (2)	C31—N4—C28—C27	−178.9 (2)
C11—C12—C13—C14	1.4 (4)	C27—C28—C29—C30	0.0 (3)
N2—C12—C13—C14	−179.4 (2)	N4—C28—C29—C30	−179.6 (2)
C10—C9—C14—C13	−0.6 (4)	C28—C29—C30—C25	0.0 (3)
S1—C9—C14—C13	−180.0 (2)	C26—C25—C30—C29	0.2 (3)
C12—C13—C14—C9	−0.4 (4)	S2—C25—C30—C29	177.16 (17)
C12—N2—C15—O3	0.5 (4)	C28—N4—C31—O6	−3.2 (4)
C12—N2—C15—C16	−179.0 (2)	C28—N4—C31—C32	175.9 (2)

### Hydrogen-bond geometry (Å, °)

**Table:**

*D*—H···*A*	*D*—H	H···*A*	*D*···*A*	*D*—H···*A*
N1—H1n···O3^i^	0.86 (2)	2.07 (2)	2.909 (3)	164 (2)
N2—H2n···O4^ii^	0.88 (2)	2.08 (2)	2.931 (3)	162.6 (19)
N3—H3n···O1^iii^	0.870 (19)	2.107 (19)	2.960 (3)	166 (2)
N4—H4n···O5^iii^	0.869 (19)	2.18 (2)	3.036 (2)	169.5 (19)
C8—H8b···O6^iv^	0.96	2.71	3.640 (3)	163
C6—H6···O6^iv^	0.93	2.67	3.541 (3)	156
C16—H16c···O6^v^	0.96	2.65	3.477 (3)	145

Symmetry codes: (i) *x*+1, *y*, *z*; (ii) −*x*+1, −*y*+1, −*z*+1; (iii) *x*−1, *y*, *z*; (iv) *x*+1, *y*+1, *z*; (v) *x*, *y*+1, *z*.
